# Protecting migratory farmers in rural Tanzania using eave ribbons treated with the spatial mosquito repellent, transfluthrin

**DOI:** 10.1186/s12936-019-3048-8

**Published:** 2019-12-10

**Authors:** Johnson K. Swai, Arnold S. Mmbando, Halfan S. Ngowo, Olukayode G. Odufuwa, Marceline F. Finda, Winifrida Mponzi, Anna P. Nyoni, Deogratius Kazimbaya, Alex J. Limwagu, Rukiyah M. Njalambaha, Saidi Abbasi, Sarah J. Moore, Joanna Schellenberg, Lena M. Lorenz, Fredros O. Okumu

**Affiliations:** 1 Environmental Health and Ecological Science Department, Ifakara Health Institute, P.O. Box 53, Ifakara, Tanzania; 2 Institute of Biodiversity, Animal Health and Comparative Medicine, University of Glasgow, Glasgow, G12 8QQ UK; 3 School of Public Health, Faculty of Health Sciences, University of the Witwatersrand, Johannesburg, South Africa; 4 Swiss Tropical and Public Health Institute, Socinstrasse. 57, 4002 Basel 4, Switzerland; 5 University of Basel, St. Petersplatz 1, 4002 Basel, Switzerland; 6 Department of Disease Control, Faculty of Infectious and Tropical Disease, London School of Hygiene and Tropical Medicine, Keppel Street, London, WC1E 7HT UK; 7 School of Life Science and Bioengineering, The Nelson Mandela African, Institution of Science and Technology, P. O. Box 447, Arusha, Tanzania

**Keywords:** Migratory rice farmers, Eave ribbons, Transfluthrin, Spatial repellent

## Abstract

**Background:**

Many subsistence farmers in rural southeastern Tanzania regularly relocate to distant farms in river valleys to tend to crops for several weeks or months each year. While there, they live in makeshift semi-open structures, usually far from organized health systems and where insecticide-treated nets (ITNs) do not provide adequate protection. This study evaluated the potential of a recently developed technology, eave ribbons treated with the spatial repellent transfluthrin, for protecting migratory rice farmers in rural southeastern Tanzania against indoor-biting and outdoor-biting mosquitoes.

**Methods:**

In the first test, eave ribbons (0.1 m × 24 m each) treated with 1.5% transfluthrin solution were compared to untreated ribbons in 24 randomly selected huts in three migratory communities over 48 nights. Host-seeking mosquitoes indoors and outdoors were monitored nightly (18.00–07.00 h) using CDC light traps and CO_2_-baited BG malaria traps, respectively. The second test compared efficacies of eave ribbons treated with 1.5% or 2.5% transfluthrin in 12 huts over 21 nights. Finally, 286 farmers were interviewed to assess perceptions about eave ribbons, and their willingness to pay for them.

**Results:**

In the two experiments, when treated eave ribbons were applied, the reduction in indoor densities ranged from 56 to 77% for *Anopheles arabiensis*, 36 to 60% for *Anopheles funestus*, 72 to 84% for *Culex,* and 80 to 98% for *Mansonia* compared to untreated ribbons. Reduction in outdoor densities was 38 to 77% against *An. arabiensis*, 36 to 64% against *An. funestus,* 63 to 88% against *Culex*, and 47 to 98% against *Mansonia*. There was no difference in protection between the two transfluthrin doses. In the survey, 58% of participants perceived the ribbons to be effective in reducing mosquito bites. Ninety per cent were willing to pay for the ribbons, the majority of whom were willing to pay but less than US$2.17 (5000 TZS), one-third of the current prototype cost.

**Conclusions:**

Transfluthrin-treated eave ribbons can protect migratory rice farmers, living in semi-open makeshift houses in remote farms, against indoor-biting and outdoor-biting mosquitoes. The technology is acceptable to users and could potentially complement ITNs. Further studies should investigate durability and epidemiological impact of eave ribbons, and the opportunities for improving affordability to users.

## Background

Malaria control has gained significant momentum in the past decade due to key interventions, including insecticide-treated nets (ITNs), indoor residual spraying (IRS), prompt diagnosis, improved chemotherapy and chemoprophylaxis, and health education [1–3]. However, the progress observed since 2000 has begun stagnating. The 2018 World Health Organization (WHO) Report indicated an increase in malaria cases in several African countries compared to the previous year [4]. This situation has been attributed to multiple challenges, such as increased propensity of malaria mosquitoes to bite outdoors and in early evenings, as well as resistance to common public health insecticides, notably pyrethroids [5–8]. Other challenges include high costs of alternative protective measures and sub-optimal user compliance. Complementary technologies are therefore required to ensure that current gains are maintained [9], but also to accelerate efforts towards set global targets [10].

Evidence suggests that improving house designs reduces the burden of vector-borne diseases [11–14]. Since 2000, economic transitions have led to rapid improvement of housing in Africa, including the doubling of families living in houses made of finished materials and with proper sanitation [15]. An increasing number of African houses now have concrete or brick walls, corrugated iron or tiled roofs, as well as screened windows and eave spaces [15]. Unfortunately, many migratory communities, such as pastoralists and seasonal rice farmers, have not benefitted from these house improvements due to low income levels and competing priorities.

**Fig. 1:**
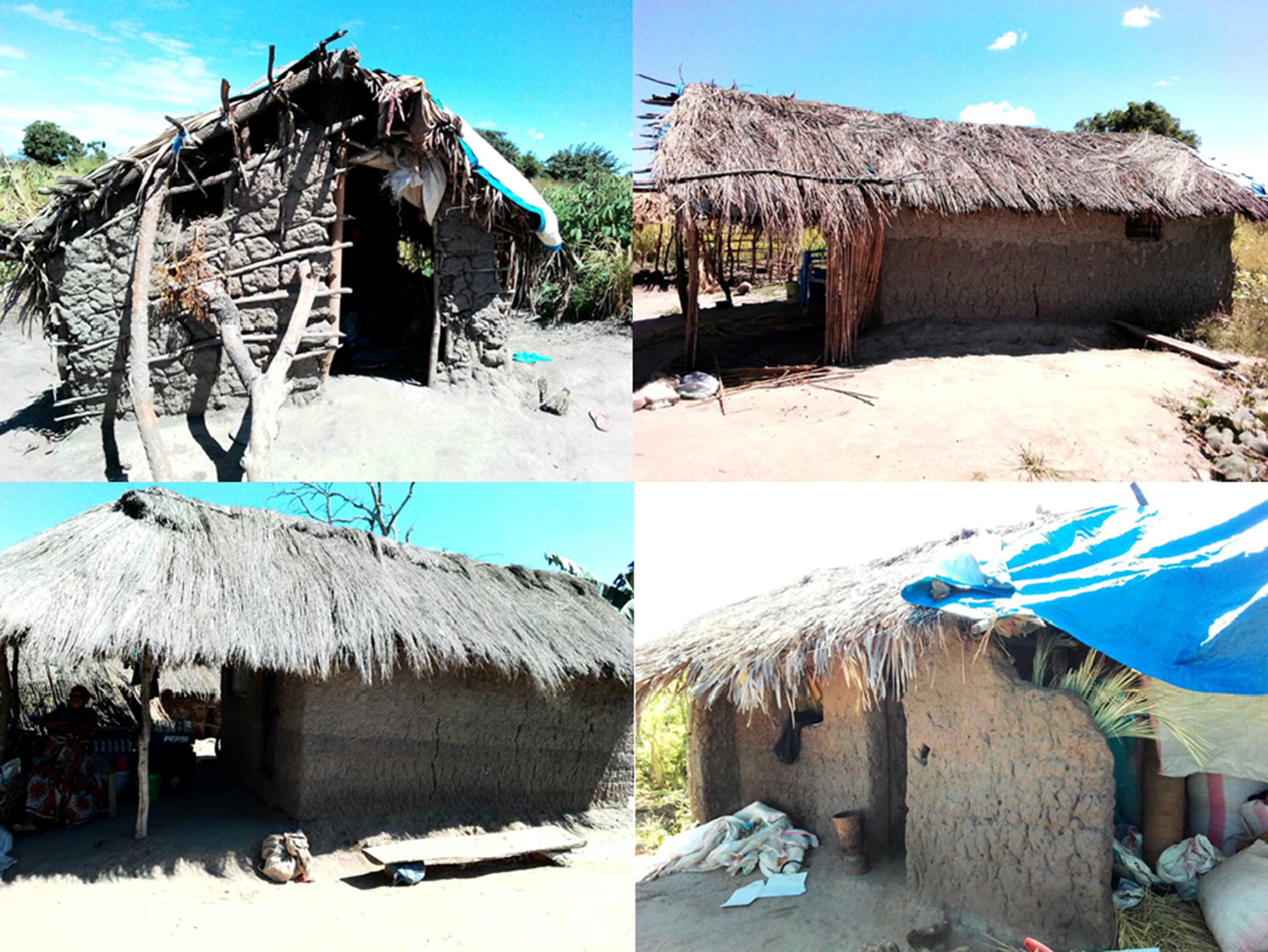
Examples of makeshift housing structures that the migratory farmers use when they go to the farms in the distant river valleys

In rural southeastern Tanzania where subsistence rice cultivation is a common economic activity, families often relocate to their farms in distant river valleys for weeks or months (usually between January and June), to tend to their crops, only returning to their main residential homes at the end of the farming season. In most cases, parents bring with them their children below school age. While at the farms, these families usually dwell in temporary structures (Fig. 1), leaving them disproportionately more exposed to mosquito bites than the rest of the population [16]. In some of these structures, use of standard prevention tools, such as ITNs, is also compromised [16]. Affordable alternatives are therefore necessary to sustainably protect these communities from potential infectious and nuisance arthropod bites.

Interventions, such as larval source management, insecticide-treated clothes, blankets and hammocks, as well as topical repellents, may constitute alternatives for such communities [9], but could be affected by poor regular compliance, high costs and poor access [17–22]. Passive spatial repellent products may offer a practical alternative. Examples include hessian materials treated with the spatial repellent, transfluthrin, which can provide long-lasting protection due to high retention of transfluthrin by the hessian fabric [23–26]. Such products typically protect more than one individual by deterring mosquitoes, inhibiting blood-feeding and killing vectors [27–31], without requiring any external energy sources or re-application of active ingredient [23, 32–34]. They can also be delivered in a variety of formats for use both indoors and outdoors. Recent studies in Tanzania used the same fabric to create transfluthrin-treated eave ribbons, which when fitted along open eave spaces of houses can effectively prevent mosquito bites both indoors and outdoors [35, 36].

This current study evaluated efficacy of transfluthrin-treated eave ribbons in protecting rural migratory farmers in their semi-open hut structures in distant rice fields in the Kilombero Valley, southeastern Tanzania. The study also assessed perceptions and willingness to pay for this tool by farmers.

## Methods

### Study area

**Fig. 2:**
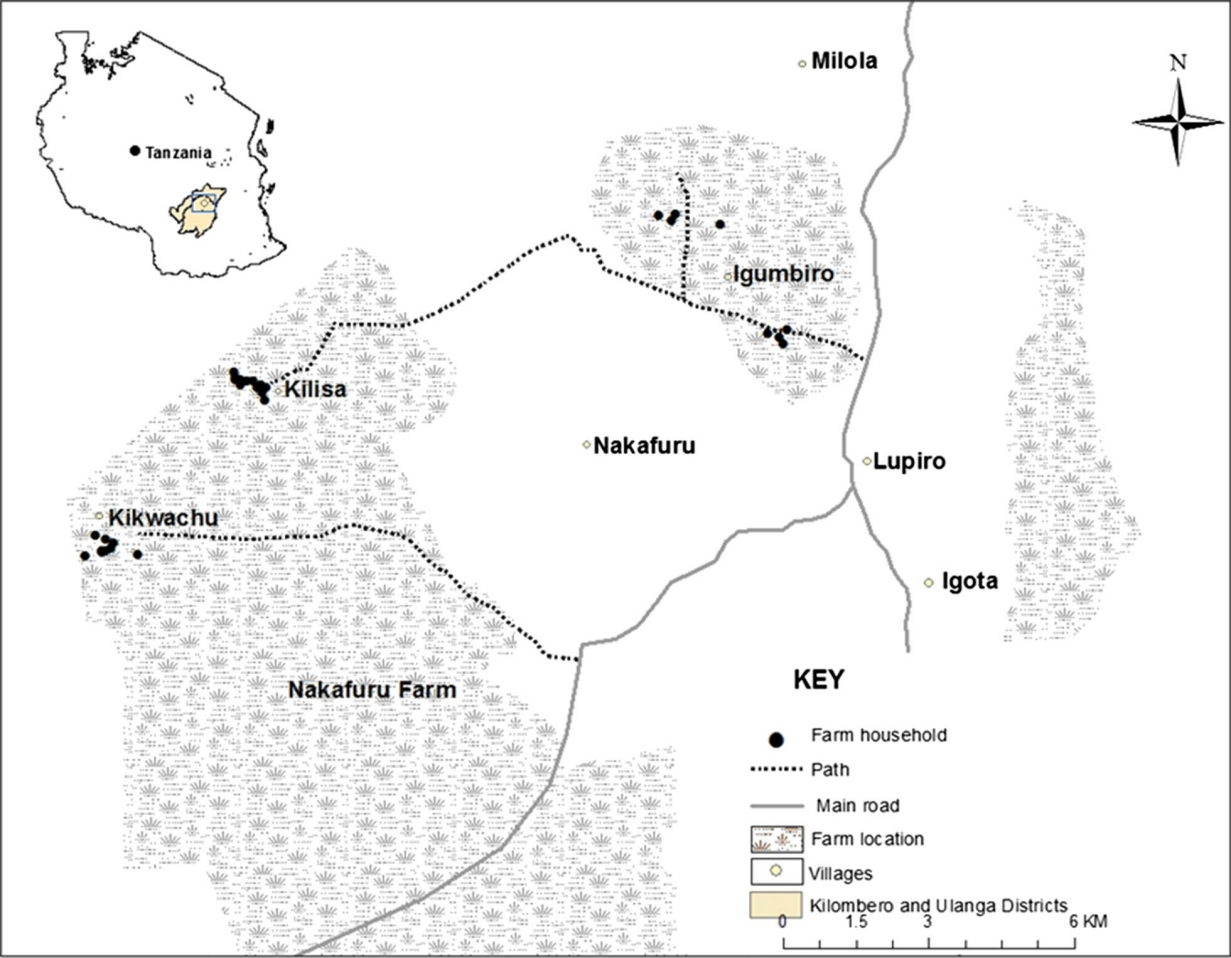
Map showing the study villages and locations of the migratory farming households that participated in the study

The study was conducted in three rice farm areas: Igumbiro, Kikwachu and Kilisa, in Ulanga district, southeastern Tanzania (Fig. 2), from March to July 2018. Annual rainfall and mean daily temperatures in the area range from 1200 to 1800 mm and 20 to 32.6 °C, respectively. The main malaria vectors include *Anopheles arabiensis* and *Anopheles funestus,* both of which are resistant to pyrethroids [37–40], and the latter mediating most of the transmission. ITNs are the main vector control tool in the region, and are often distributed by the Government to the main residential communities [41]. Although compliance to ITN use in farmhouses is high [42], these rice farmers remain at risk of nuisance and potential infective bites before going under a bed net due to the poor housing structures that allow easy entry of mosquitoes.

A recent cross-sectional survey across residential villages in Kilombero and Ulanga found the prevalence varies between < 1% in peri-urban and > 40% in rural villages that are ~ 20 km away from the rice farms where the study was conducted (Swai et al., unpublished).

### Transfluthrin-treated eave ribbons

**Fig. 3:**
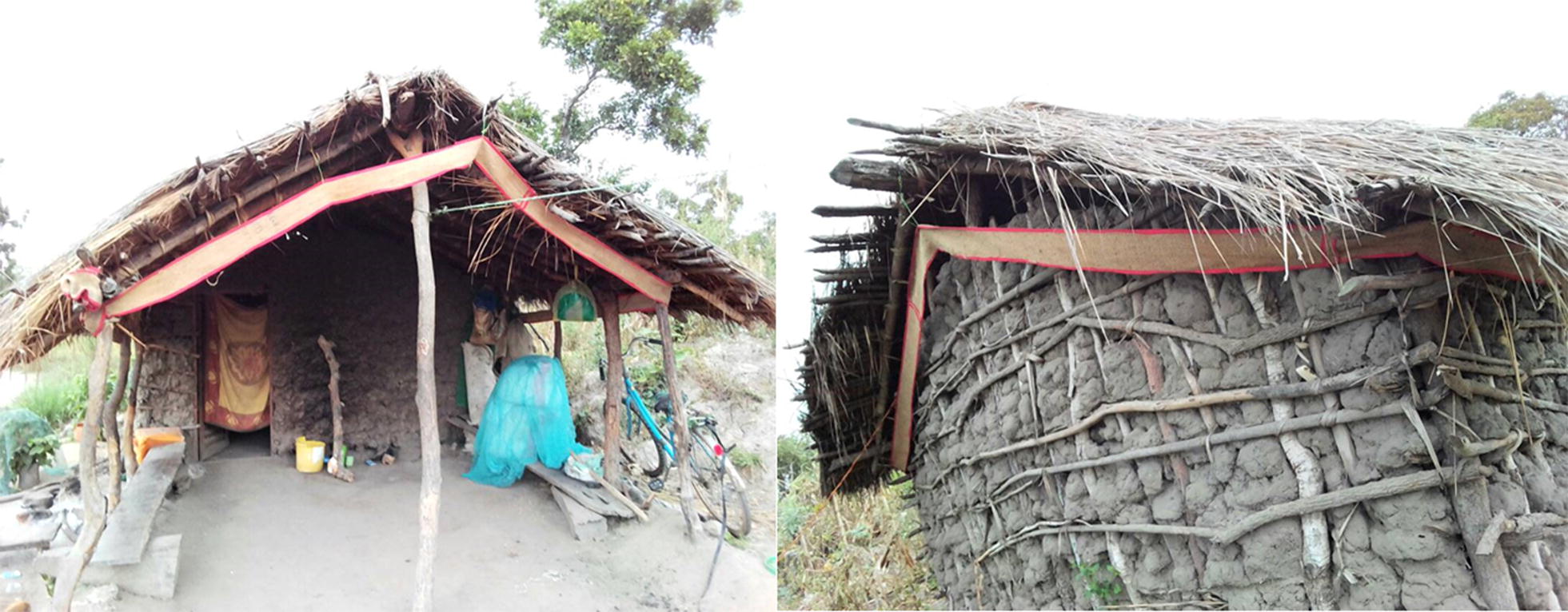
Selected structures for evaluating the use of transfluthrin-treated eave ribbons installed along the eaves spaces of farm huts for preventing mosquito bites indoors and outdoors

The eave ribbons were prepared as described by Mmbando et al. [35] and cut to size of 0.1 m wide and 24 m long, so that they could fit all around the farm huts (Fig. 3). Treatment of the eave ribbons also followed published procedures [23, 25, 35]. To achieve 1.5 and 2.5% transfluthrin concentrations, 22.5 ml and 37.5 ml of technical grade transfluthrin stock solution were dissolved in 127.5 ml and 112.5 ml of liquid detergent (Axion^®^), respectively, and 1350 ml of water added, so that the total volume was always 1500 ml. The control eave ribbons were prepared by soaking the ribbons in a mixture of 150 ml Axion^®^ liquid detergent and 1350 ml water, without any transfluthrin. Thus, for the 1.5 and 2.5% concentrations, the amount of transfluthrin per surface area was 13.73 g/m^2^ and 22.89 g/m^2^, respectively.

### Assessing efficacy of 1.5% transfluthrin-treated eave ribbons

Using a randomized cross-over design with a wash period of 2 days between treatments, entomological efficacy of eave ribbons treated with 1.5% transfluthrin solution was evaluated as follows: from each of the three rice farms, 8 huts from consenting rice farmers were recruited, so that there was a total of 24 huts. Of the 8 huts in each village, 4 huts were randomly allocated the transfluthrin-treated eave ribbons, while the remaining 4 received untreated eave ribbons (controls). The eave ribbons were fitted along the eave spaces every evening by trained technicians and removed each morning (Fig. 3). The treatment arms were rotated after every 4 consecutive nights of trapping. The experiment was conducted over a total of 16 nights in each village, during which each hut received both the transfluthrin-treated and control eave ribbons twice.

To minimize cross contamination between the treated and control ribbons, the ribbons were fitted in such a way as to ensure no physical contact with the walls or roof of the hut. In addition, a wash out period of 2 days was implemented between rotations, during which no ribbons were fitted to the huts and no experiments were done. All households participating in the study were provided with a new ITN, i.e., Olyset^®^ net (A to Z Textiles, Arusha, Tanzania), as basic protection during sleeping hours.

Each night, indoor and outdoor mosquito collections were done between 18.00 and 07.00 h using CDC^®^ light traps (CDC-LT) [43] and carbon dioxide-baited BG^®^ malaria traps (BGM) [44, 45], respectively. Each morning, the collected mosquitoes were sorted by sex, and all female mosquitoes were morphologically identified into taxa following taxonomic keys [46, 47], and grouped as blood-fed, unfed or gravid. The female *Anopheles* were preserved in silica-filled microcentrifuge tubes then processed by polymerase chain reaction (PCR) and enzyme-linked immunosorbent assays (ELISA) to respectively distinguish between sibling species [48–50] and detect any *Plasmodium* infections in the salivary glands [51]. Outdoor temperature and humidity were recorded using Tiny tag^®^ data recorders that were hung away from direct sunlight and rain.

### Assessing efficacy of 2.5% transfluthrin-treated eave ribbons

Entomological efficacy of 2.5% transfluthrin-treated eave ribbons was evaluated using a three-by-three Latin-square experimental design with a wash out period of 2 days between treatments. Twelve farm huts from consenting migratory rice farmers in Kilisa village were recruited. Four of the huts received eave ribbons treated with 1.5% transfluthrin solution, another 4 huts received ribbons with 2.5% transfluthrin solution and the remaining 4 were fitted with untreated ribbons (i.e., controls). The ribbons were fitted along the eave spaces as described above, and all huts were provided with new Olyset^®^ nets for basic protection. The treatments were rotated between the huts every week. Unfortunately, due to a government directive for the farmers to vacate the fields, the experiment was terminated after 21 nights only. Mosquitoes were processed and environmental data were collected as described above.

### Identification of sibling species, blood meals and *Plasmodium* infections in malaria vectors

The PCR assays for sibling species of *Anopheles gambiae* sensu lato (*s.l.*) were performed as described by Scott et al. [48], while those for *An. funestus* group were done following Koekemoer et al. [49] and Cohuet et al. [50] techniques. All blood-fed *Anopheles* mosquitoes were screened for human, bovine, chicken, goat, and dog blood using ELISA [51]. Additionally, all female *Anopheles* were assessed for *Plasmodium* circumsporozoite proteins using ELISA (csp-ELISA). To eliminate false positives, which are heat labile, all csp-ELISA positive lysates were boiled for 10 min at 100 °C and re-tested [52].

### Assessing community perceptions and their willingness to pay for transfluthrin-treated eave ribbons

A quantitative survey was conducted after completion of the entomological efficacy assays to examine the farmers’ perceptions and willingness to pay for the transfluthrin-treated eave ribbons. A total of 286 individuals from the 3 rice farms were recruited. The actual number of individuals from each farm area was proportional to the population size of the area, thus 10, 114 and 162 individuals were recruited from Igumbiro, Kilisa and Kikwachu, respectively. A list of names of farmers was sought from respective local administrative leaders in the rice farms. These were then entered into Excel, random number generated for each farmer and, following systematic sample selection procedures, farmers were randomly selected and recruited. Since most farmers had already vacated their fields at the time of this survey, the interviews were conducted with the respondents at their main residential homes rather than the farms.

A structured questionnaire was administered to collect information on basic demographic and socio-economic traits. Participants were also asked about the current mosquito control tools used while at the rice farms, how much they spent on mosquito control the previous year, their perceptions of eave ribbons as a control tool, and their willingness to pay for the transfluthrin-treated eave ribbons. Willingness to pay was assessed by asking whether participants were willing to purchase the treated eave ribbons, and amount of money they were willing to spend in Tanzanian shillings (TZS). Before asking these questions, the interviewers showed samples of untreated eave ribbons, and explained to the participants how the eave ribbons function.

### Data analysis

Mosquito count data were analysed using Stata^®^ 13 (College Station, TX, USA). The effects of treatment on indoor and outdoor mosquito densities in different study arms were examined using generalized linear mixed effects models (GLMMs) with a negative binomial distribution and log-link function to account for overdispersion of the data. Mosquito densities were modelled as a function of treatment with household ID, day and village as random factors. Protective efficacy of the eave ribbons with different transfluthrin concentrations were calculated from the relative risk (RR) values for the control (C) and treatment (T) arms in the no-intercept model, using the formula (C-T)/C*100. Blood meal sources and *Plasmodium* infections rates were calculated as percentages of total mosquitoes assayed.

Entomological inoculation rates (EIR), i.e., number of malaria infectious bites per person per given period, were determined by multiplying average nightly biting rates and the proportion of sporozoite positive mosquitoes. The nightly biting rate was calculated by dividing total mosquitoes caught by product of number of households and trap nights. To estimate annual EIR, the daily EIR values were multiplied by 365 nights (annual EIR = nightly biting rate × sporozoite rate × 365). Adjusted EIR were calculated by multiplying estimated EIR by correction factors from previous evaluations comparing CDC-LT and BGM trap catches to actual number of mosquitoes that bite unprotected human volunteers, i.e., in human landing catches [53, 54]. A power calculation with GLM simulation modelling using R statistical software [55, 56] confirmed that both experiments were adequately powered (> 80%) despite the second one running for just 21 days.

The perceptions and willingness to pay for the eave ribbon data was analysed as follows: socio-economic status of individuals was first estimated using principal component analysis (PCA), where household assets, structure, energy and water sources were used to classify individuals among the study population into five quintiles. The purchasing amounts mentioned by the participants were grouped into two categories US$ < 2.17 and ≥ 2.17 [(< 5000 and ≥ 5000 TZS) as per exchange rates of January 2019]. Descriptive analysis was conducted to determine the respondent’s perception of effectiveness, willingness to pay by gender, socio-economic status, education levels, and marital status. Perception of effectiveness was summarized using percentages. Pearson’s Chi square test and Chi square test for trend were used to assess association between socio-economic status, frequencies of willingness to pay and the purchasing amount quoted by the respondents. Finally, associations between socio-economic status and quoted purchasing amount was assessed using multivariate logistic regression analysis controlling for age, education and amount of money spent on malaria control the previous year.

## Results

### Protective efficacy of eave ribbons treated with 1.5% transfluthrin

A total of 3872 *An. arabiensis*, 1232 *An. funestus*, 495 *Anopheles coustani*, 1200 *Anopheles pharoensis*, 3780 *Culex* spp. 2234 *Mansonia* spp. and 97 *Aedes* spp. female mosquitoes were caught in the 3 rice farms. Igumbiro village had the highest densities of *An. funestus* and *Culex* spp. while Kikwachu and Kilisa had the highest densities of *An. arabiensis* and *Mansonia* spp. Kilisa also had the highest densities of *Anopheles coustani* and *Anopheles pharoensis* (Table 1).

**Table 1:** Biting densities of different mosquito species caught in the three study rice farms during the study period

Mosquito species	Igumbiro, n (%)	Kikwachu, n (%)	Kilisa, n (%)
*Anopheles arabiensis*	447 (11)	1876 (39)	2448 (18)
*Anopheles funestus*	901 (21)	87 (2)	5025 (38)
*Anopheles coustani*	31 (1)	82 (2)	761 (6)
*Anopheles pharoensis*	_	186 (4)	1762 (13)
*Culex* species	2255 (53)	643 (13)	993 (8)
*Mansonia* species	570 (13)	1869 (39)	2219 (16)
*Aedes* species	32 (1)	60 (1)	30 (1)
Total	4236 (100)	4803 (100)	13,238 (100)

Eave ribbons treated with 1.5% transfluthrin reduced indoor densities of *An. arabiensis* by 56% (p < 0.001), *An. funestus* by 36% (p < 0.001), *Culex* spp. by 72% (p < 0.001) and *Mansonia* spp. by 80% (p < 0.001). The ribbons also reduced outdoor biting for *An. arabiensis* by 38% (p = 0.034)*, Culex* spp. by 64% (p < 0.001) and *Mansonia* spp. 47% (p < 0.001), respectively (Table 2). There was no observable protection against *An. funestus* outdoors. Over the duration of the experiment, the nightly temperatures ranged from 22.5 to 31.9 °C, averaging at 25.2 °C, while percentage relative humidity ranged from 65.6 to 100%, averaging at 91.9%.

**Table 2:** Protection conferred indoors and outdoors against anopheline and culicine species in all three rice farms when 1.5% transfluthrin-treated eave ribbons were fitted along the eaves of rice farm huts

Mosquito species	Treatment	Indoors				Outdoors			
		Total number	Geometric mean (LCI-UCI)	% protection (LCI-UCI)	p value	Total number	Geometric mean (LCI-UCI)	% Protection (LCI-UCI)	p value
*Anopheles arabiensis*	Untreated	2526	9.4 (8.0–11.0)	–	< 0.001	135	1.7 (1.4–2.0)	–	0.034
	1.5% transfluthrin	1131	4.8 (4.2–5.5)	56 (47–64)		80	1.3 (1.1–1.6)	38 (3–60)	
*Anopheles funestus*	Untreated	751	3.0 (2.5–3.6)	–	0.001	21	1.2 (1.0–1.5)	–	0.413
	1.5% transfluthrin	445	2.4 (2.1–2.8)	36 (16–50)		15	1.2 (1.0–1.5)	–	
*Culex* species	Untreated	2986	7.8 (6.5–9.3)	–	< 0.001	100	2.0 (1.6–2.5)	–	0.003
	1.5% transfluthrin	656	3.2 (2.8–3.7)	72 (65–77)		38	1.3 (1.1–1.5)	62 (29–80)	
*Mansonia* species	Untreated	3510	12.9 (10.8–15.5)	–	< 0.001	290	2.3 (1.9–2.7)	–	0.001
	1.5% transfluthrin	628	3.8 (3.2–4.4)	80 (75–85)		150	1.8 (1.5–2.2)	49 (24–66)	

Each treatment arm had 48 nights of sampling. Percentage protective efficacy is estimated for each treatment relative to the respective controls

*LCI* lower confidence interval, *UCI* upper confidence interval, *p value* Walds p value

### Protective efficacy of eave ribbons treated with 2.5% transfluthrin

In tests comparing the eave ribbons treated with either 1.5 or 2.5% transfluthrin, 899 *An. arabiensis,* 4781 *An. funestus*, 379 *Culex,* 748 *Mansonia*, and 25 *Aedes* mosquitoes were caught. Both 1.5 and 2.5% transfluthrin-treated eave ribbons conferred substantial protection indoors and outdoors against all mosquito taxa. The highest protection was 76 to 98% against *Mansonia* spp. followed by 63 to 88% protection against *Culex* spp. 72 to 77% against *An. arabiensis*, and 59 to 64% against *An. funestus*, compared to untreated control ribbons (Table 3). There was no significant difference in protection offered by the two doses. The nightly temperatures ranged between 18.8 and 30.8 °C, averaging at 23.2 °C, while relative humidity ranged between 53.8 and 100%, averaging at 87.4%.

**Table 3:** Protection conferred indoors and outdoors against anopheline and culicine species in Kilisa rice farms when 1.5 and 2.5% transfluthrin-treated eave ribbons were fitted along the eaves of rice farm huts

Mosquito species	Treatment	Indoors				Outdoors			
		Total	Geometric mean (LCI-UCI)	% protection (LCI-UCI)	p values	Total	Geometric mean (LCI-UCI)	% protection (LCI-UCI)	p values
*Anopheles arabiensis*	Untreated	497	5.00 (4.20–5.94)	_	_	58	1.57 (1.31–1.90)	_	_
	1.5% transfluthrin	148	2.12 (1.70–2.64)	77 (64–85)	< 0.001	16	1.10 (0.95–1.28)	77 (48–90)	< 0.001
	2.5% transfluthrin	163	2.20 (1.74–2.78)	74 (59–84)	< 0.001	17	1.30 (1.00–1.69)	72 (37–88)	0.002
*Anopheles funestus*	Untreated	2093	20.71 (17.96–23.89)	_	_	479	4.30 (3.44–5.39)	_	_
	1.5% transfluthrin	868	9.84 (8.47–11.44)	60 (49–68)	< 0.001	184	2.18 (1.82–2.60)	56 (39–68)	< 0.001
	2.5% transfluthrin	988	10.85 (9.14–12.89)	60 (49–68)	< 0.001	169	2.25 (1.90–2.66)	59 (44–71)	< 0.001
*Culex* species	Untreated	282	3.09 (2.54–3.75)	_	_	12	1.22 (0.89–1.67)	_	_
	1.5% transfluthrin	44	1.55 (1.23–1.96)	84 (73–90)	< 0.001	4	1 (1–1)	63 (0 –91)	0.187
	2.5% transfluthrin	32	1.55 (1.20–1.99)	88 (79–93)	< 0.001	5	1.44 (0.30–6.97)	69 (0–94)	0.124
*Mansonia* species	Untreated	664	7.51 (6.31–8.94)	_	–	25	1.70 (1.26–2.31)	_	_
	1.5% transfluthrin	14	1.36 (0.92–2.00)	98 (96–99)	< 0.001	10	1.51 (0.92–2.50)	76 (0–97)	0.145
	2.5% transfluthrin	29	2.22 (1.29–3.83)	97 (93–98)	< 0.001	6	1.32 (0.55–3.15)	90 (25–99)	0.026

Each treatment arm had 21 nights of sampling. Percentage protective efficacy is estimated for each treatment relative to the respective controls

*n* number of nights, *LCI* lower confidence interval, *UCI* upper confidence interval, *p value* Wald’s p value

### Malaria vector sibling species and blood meal sources

A total of 4771 *An. gambiae* s.l. and 6013 *An. funestus* s.l. mosquitoes underwent species identification by PCR, and blood meal analysis and circumsporozoite protein ELISA assays. All *An. gambiae* s.l were found to be *An. arabiensis* (100%), while the *An. funestus* group consisted of 91.4% *An. funestus* sensu stricto (s.s.), 2.9% *Anopheles rivolurum*, 0.9% *Anopheles leesoni*, and 5.8% unamplified samples. Twenty-three mosquitoes blood-fed were caught (14 *An. arabiensis* and 9 *An. funestus* s.s.). Blood meal ELISA revealed that 2 (14%), 5 (36%) and 7 (50%) of the *An. arabiensis* had fed on human, bovine and goat, respectively, while all *An. funestus* s.s. had fed on humans.

### *Plasmodium* sporozoite infection rates and malaria transmission intensities

Overall, malaria transmission in the rice farms was low; 14 mosquitoes were found with *Plasmodium* sporozoites (one *An. arabiensis,* 13 *An. funestus* s.s.). Four of the *An. funestus* were caught outdoors while the rest, including the *An. arabiensis,* were caught indoors. EIR for *An. arabiensis* and *An. funestus* were estimated separately for both indoor and outdoor environments, from the CDC-LT and BGM trap catches, respectively (Table 4). Infection rates were higher in *An. funestus* than *An. arabiensis* both indoors and outdoors, and *An. funestus* was responsible for more than 90% of all transmission occurring in the area. Most transmission occurred indoors, where *An. funestus* had an annual EIR estimate of 2.33 infectious bites per person per year (ib/p/y) indoors compared to 0.79 ib/p/y outdoors. However, since farmers typically spend no more than half a year in the farms, actual infection intensities would be less than half of these estimates.

**Table 4:** Infectiousness of *Anopheles arabiensis* and *Anopheles funestus* mosquitoes caught indoors and outdoors the rice farms

	Indoors		Outdoors	
	*Anopheles arabiensis*	*Anopheles funestus*	*Anopheles arabiensis*	*Anopheles funestus*
Total mosquitoes analysed	4465	5145	306	868
Number of households	24	24	24	24
Number of trapping nights	69	69	69	69
Mosquito/household/night	2.70	3.11	0.18	0.52
Corrected biting rate	3.53	3.29	0.03	0.63
*Plasmodium* positive mosquitoes	1	10	0	3
Sporozoite rate	0.0002	0.0019	0	0.0035
EIR	0.0006	0.0060	0	0.0018
Annual EIR	0.2204	2.2041	0	0.6612
Corrected EIR	0.0008	0.0064	0	0.0022
Corrected Annual EIR^a^	0.2887	2.3363	0	0.7935
EIR contribution indoors and outdoors	9%	91%	0	100%
Corrected EIR contribution indoors and outdoors	4%	96%	0	100%
Overall EIR contribution	7%	71%	0	22%
Overall corrected EIR contribution	3%	64%	0	33%

Corrected biting rate indoor **= **mosquito/household/trap × relative efficacy of CDC-LT to HLC [53], i.e., 0.3 for *An. arabiensis* and 0.68 for *An. funestus* Corrected biting rate outdoor = mosquito/household/trap × relative efficacy of BGM to HLC [54], i.e., 0.16 for *An. arabiensis* and 1.2 for *An. funestus*

^a^Since farmers typically spend less than half a year in the farms, actual infection intensities would be less than half of these estimates

### Perception of community members regarding the mosquito bites, malaria and eave ribbons

Of the 286 respondents, 92% were subsistence farmers engaged in crop cultivation, while the rest engaged in multiple activities, such as business and fishing in addition to farming. Age of respondents ranged between 18 and 80 years, with the median age being 38. Most respondents (82%; n = 235) had primary level education and 75% (n = 215) were living with a partner. Only 31% (n = 89) of the respondents’ main residential homes had screened windows, 77% (n = 220) had brick walls, 69% (n = 197) had roofs made of iron sheets, and 27% (n = 78) had modern toilet facilities (flush or improved ventilated pit latrines). Almost three-quarters (72%; n = 206) of the respondents had access to piped water, mostly from communal boreholes.

Over 80% of the respondents reported that malaria is a major concern in the rice farms. Most (90%; n = 257) used bed nets for protection against mosquito bites when at the farms. They also reported using non-residual insecticide sprays (mostly pyrethrum), topical repellents, mosquito coils, and smoke from burning wood. Regarding cost for malaria prevention and treatment per year, about one-quarter of the participants (23%; n = 66) said they incurred no cost, 12% (n = 33) spent less than US$2.17 (5000 TZS), 46% (n = 134) spent between US$2.60 and 4.33 (6000-10,000 TZS), 12% (n = 32) between US$4.77 and 8.66 (11,000-20,000 TZS), and 5% spent more than US$9.11 (> 21,000 TZS).

Nearly two-thirds of participants (60%; n = 172) reported facing challenges in protecting themselves or their families against mosquito bites while at the farms. The commonest challenges included: (a) bed nets having large holes, letting in mosquitoes or mosquitoes biting through the nets; (b) high costs and low access of insecticide sprays and topical repellents; (c) lack of effective outdoor protection tools; and, (d) canned insecticide sprays not lasting long.

### Current expenditures on malaria prevention, and people’s willingness to pay for transfluthrin-treated eave ribbons

More than 90% of participants said that they would use and pay for the transfluthrin-treated eave ribbons if available. When asked how much they were willing to pay, 70% (n = 200) stated values less than US$2.17 (5000 TZS) for the intervention, while 23% (n = 66) were willing to pay between US$2.21 and 4.33 (5100–10,000 TZS).

Although there was no evidence for association between socio-economic status and willingness to pay for the ribbons (Fisher’s exact test p = 0.558), socio-economic status strongly influenced whether individuals were willing to pay more or less than a set cut-off of US$2.17 (5000 TZS) (Fisher’s exact test p = 0.016). The least poor were more than three times more likely to be willing to pay above this cut-off [(OR = 3.2; 95% CI (1.2–8.3) p = 0.0139]. In addition, individuals who spent US$ > 2.17 per year on mosquito prevention tools were more likely to pay more for the eave ribbons (Table 5). After adjusting for age, education level and amount of money generally spent on mosquito prevention tools, only individuals who spend US$ > 2.17/year on mosquito prevention were willing to pay US$ > 2.17 for the eave ribbons (Table 5).

**Table 5:** Factors affecting the amount individuals are willing to pay for the transfluthrin-treated eave ribbons

Characteristic	Level	Unadjusted OR (LCI-UCI)	p value	Adjusted OR (LCI-UCI)	p value
Socio-economic status	Poorest	_	_	_	_
	Poor	1.3 (0.5–3.7)	0.564	1.0 (0.3–3.2)	0.932
	Middle	1.1 (0.4–3.2)	0.825	0.9 (0.3–2.8)	0.847
	*Less poor*	*3.1 (1.2*–*7.9)*	*0.019*	2.7 (0.9–7.7)	0.066
	*Least poor*	*3.2 (1.2*–*8.1)*	*0.016*	2.5 (0.9–7.3)	0.089
Age (in years)	18–24	_	_	_	_
	25–29	1.1 (0.4–3.4)	0.811	1.5 (0.4–5.7)	0.523
	30–34	0.4 (0.1–1.5)	0.200	0.5 (0.1–2.2)	0.367
	35–39	1.0 (0.3–3.0)	1.000	1.1 (0.3–4.2)	0.861
	40–44	1.4 (0.4–4.1)	0.600	1.6 (0.4–6.8)	0.495
	45–49	0.8 (0.2–3.2)	0.711	1.7 (0.3–9.0)	0.557
	50–54	0.3 (0.1–1.5)	0.163	0.5 (0.1–2.8)	0.405
	55–59	0.7 (0.2–3.0)	0.629	0.7 (0.1–3.8)	0.640
	60–64	0.5 (0.1–3.0)	0.453	1.4 (0.2–10.7)	0.771
	65+	0.5 (0.1–2.1)	0.343	0.7 (0.1–3.7)	0.630
Education	None/primary	_	_	_	_
	Secondary/higher	1.1 (0.5–2.6)	0.8477	0.8 (0.3–2.5)	0.745
Malaria control expenses (previous year)	None	_	_	_	_
	< 2.17 USD	1.6 (0.6–4.5)	0.3337	2.0 (0.7–5.9)	0.223
	> *2.17 USD*	*6.2 (3.1*–*12.3)*	< *0.001*	*6.9 (3.4*–*14.0)*	< *0.001*

Italics: Factors with evidence (p < 0.05) that they influence amount individuals are willing to pay

## Discussion

In this study, the potential of transfluthrin-treated eave ribbons as a protective measure against disease-transmitting and nuisance-biting mosquitoes was evaluated in rice farms of southeastern Tanzania where seasonal migrant farmers live in poorly constructed makeshift housing. Overall, the study results demonstrate that in addition to ITNs, transfluthrin-treated eave ribbons can be used to reduce densities of indoor-biting and outdoor-biting mosquitoes. Since the experimental controls had untreated eave ribbons, the observed reduction may be attributed to the transfluthrin-treatment, rather than any physical barrier from the hessian fabric. Earlier tests by Mmbando et al. demonstrated that eave ribbons work by both repelling host-seeking mosquitoes and directly killing those that come in contact with the vapours [35].

In particular, the reduction of mosquito bites recorded outdoors show that there is a protective radius offered by the transfluthrin-treated eave ribbons beyond the physical house structure itself [24]. Many Tanzanian families spend long periods of time outdoors in the evenings doing various activities, such as cooking, story-telling or playing (children), which exposes them to potentially infectious bites [57–59]. It is evident that transfluthrin-treated eave ribbons offer additional protection in such spaces, thereby complementing ITNs. The observed effects were apparent against the malaria vectors, *An. arabiensis* and *An. funestus*, but also non-malaria mosquitoes such as *Culex* and *Mansonia* spp. both indoors and outdoors.

The protection against *An. funestus,* which now dominates malaria transmission in the area, was modest in the first experiment but substantial in the second experiment. This is particularly interesting since both *An. funestus* and *An. arabiensis* are also known to be resistant to common public health pesticides including pyrethroids [37–40, 46]. The farms in Kilisa village in particular had very high densities of *An. funestus*, most likely because of the more permanent water sources in the area, which favour proliferation of this species [60, 61]. It is here too where efficacy of eave ribbons was highest against this species. The transfluthrin-treated eave ribbons were even more protective against culicines than anophelines (Tables 2 and 3). Considering that culicine species are usually more abundant in the area, resistant to most of the public health chemicals (Matowo et al. unpublished) and regularly enter the house via windows and doors [62], the level of nuisance biting and pathogens transmitted by culicines, such as filarial worms, could be greatly reduced by using transfluthrin-treated eave ribbons. Such applications could certainly extend beyond migratory farming communities. It is however particularly important that this technology is used in addition to, not as a replacement of existing interventions such as ITNs. The lack of evidence of a difference in protection conferred by 1.5 and 2.5% transfluthrin treatments suggest that the lower dose would be preferable. Similar treatment doses have previously shown field efficacy against bites by these vector species in experimental huts built in rural southeastern Tanzania [35].

More than 90% of the indoor and outdoor malaria transmission occurring in the rice farms was mediated by *An. funestus*. These findings match those of a recent entomological surveillance across villages in Ulanga district, Tanzania, which showed that despite their low numbers, *An. funestus* mediated more than four-fifths of malaria transmission [41]. The EIR observed in the rice farms was however lower than those observed in residential homes in the same district [41]. This is most probably because the small and transient human population in such migratory farming communities is inadequate to sustain higher malaria transmission intensities, even when environmental conditions in the rice farms support the proliferation of mosquitoes.

The risk of malaria transmission in the rice farms was also higher indoors than outdoors. EIR estimates indoors were 2.33 ib/p/y compared to 0.79 ib/p/y outdoors (for *An. funestus*). Similar observations that transmission is still mostly indoors despite increasing propensity of outdoor biting is also widely reported elsewhere in southeastern Tanzania [41] and western Kenya [63] where *An. funestus* and *An. arabiensis* are the most predominant vector species. Though the number of blood-fed specimen were few, *An. funestus* had higher human blood index than *An. arabiensis*, suggesting that even in remote areas where human presence is seasonal, *An. funestus* remains highly anthropophagic. This not only highlights the importance of improving the use of home-based interventions such as ITNs, but also the need to design better tools to protect such disfranchised communities from infectious bites, thereby accelerating the overall goal of malaria elimination. Given the protective efficacy observed during this study, transfluthrin treated eave ribbons can potentially be used as a complimentary intervention in such settings to protect against mosquito bites occurring inside and within peri-domestic areas when people are not under a bed net.

The majority of rice farmers highlighted malaria as a major health concern, especially while they are in their distant farms. Although bed nets are common, the farmers also used locally available insecticide sprays, topical repellent, mosquito coils, and smoke from burning wood. Mmbando et al. reported that the initial prototypes of the eave ribbons cost about US$7 (16,157 TZS) from production to installation [35]. However, more than two-thirds of participants were willing to purchase the product at less than or equal to US$2.17 (5000 TZS), which is just under one-third of the cost, and only individuals from the upper two socio-economic quintiles were willing to pay more than US$2.17 (5000 TZS). Interestingly, nearly all respondents said they would use the technology if produced and marketed to them. Considering that mass production of the transfluthrin-treated eaves ribbons would lower the price, it is possible that the technology could be made more affordable and therefore accessible for these communities. One limitation of this specific aspect of the study was that the method used for determining community members’ willingness to pay was focused only on prices, thus ignoring other key attributes such as aesthetic concerns and user compliance. It therefore captured only the hypothetical purchasing behaviour, and could be prone to response bias [64].

Future studies should evaluate additional effects of the product, including 24-h mortality of wild pyrethroid-resistant mosquitoes as they enter and leave the human dwellings fitted with the eave ribbons. Such additional benefits could magnify the overall protective efficacy of the technology, and potentially extend it to community benefits, as recently demonstrated in semi-field settings [36]. Additional research questions that should be addressed include: (a) whether such products can divert mosquitoes to non-users as previously demonstrated in areas of sub-optimal coverage [65]; (b) the duration of efficacy, which according to semi-field studies is up to 6 months [23], and could therefore effectively cover the entire farming season; (c) opportunities for engaging local communities to accelerate the adoption of this or other similar interventions to complement ITNs; and, (d) community-wide impact on malaria transmission and burden, when the eave ribbons are used alongside ITNs or other interventions. Fortunately, many of these studies are already planned or are ongoing.

## Conclusion

Transfluthrin-treated eave ribbons could be used in addition to ITNs to protect seasonal migratory farmers against both indoor and outdoor infectious and nuisance-biting mosquitoes, while they are at their distant farms. The technology is simple, portable, easy to use, widely acceptable, and can be readily fitted onto the makeshift huts that the farmers construct and use when at their farms. Most importantly, it does not require daily compliance as for bed nets or topical repellents. Most of the malaria transmission in the rice farms occurred indoors and was mediated by *An. funestus*. Rice farmers in the region showed willingness to use and purchase the eave ribbons as a control measure. There is a need to conduct further studies on longevity of protection conferred by the eave ribbons, epidemiological impacts on malaria and its transmission, as well as appropriate financing models for scale-up.

## Data Availability

The datasets used and/or analysed during the current study are available from the corresponding author on reasonable request.

## Abbreviations

*An.*: *Anopheles*
Spp: species
BG: biogents
CO_2_: carbon dioxide
ITN: insecticide-treated nets
IRS: indoor residual sprays
g/sq m: grams per square metre
ml: millilitres
PCR: ploymerase chain reaction
ELISA: Enzyme Linked Immunosorbent Assay
Csp: cricumsporozoite
TZS: Tanzanian Shillings
CDC: Centre of disease control
CDC_LT: Centre of disease control light traps
US $: United States of America Dollars
PCA: principal component analysis
